# Causal enhanced drug–target interaction prediction based on graph generation and multi-source information fusion

**DOI:** 10.1093/bioinformatics/btae570

**Published:** 2024-09-23

**Authors:** Guanyu Qiao, Guohua Wang, Yang Li

**Affiliations:** School of Computer Science and Technology, Harbin Institute of Technology, Harbin 150001, China; School of Computer Science and Technology, Harbin Institute of Technology, Harbin 150001, China; College of Computer and Control Engineering, Northeast Forestry University, Harbin 150040, China; College of Computer and Control Engineering, Northeast Forestry University, Harbin 150040, China

## Abstract

**Motivation:**

The prediction of drug–target interaction is a vital task in the biomedical field, aiding in the discovery of potential molecular targets of drugs and the development of targeted therapy methods with higher efficacy and fewer side effects. Although there are various methods for drug–target interaction (DTI) prediction based on heterogeneous information networks, these methods face challenges in capturing the fundamental interaction between drugs and targets and ensuring the interpretability of the model. Moreover, they need to construct meta-paths artificially or a lot of feature engineering (prior knowledge), and graph generation can fuse information more flexibly without meta-path selection.

**Results:**

We propose a causal enhanced method for drug–target interaction (CE-DTI) prediction that integrates graph generation and multi-source information fusion. First, we represent drugs and targets by modeling the fusion of their multi-source information through automatic graph generation. Once drugs and targets are combined, a network of drug–target pairs is constructed, transforming the prediction of drug–target interactions into a node classification problem. Specifically, the influence of surrounding nodes on the central node is separated into two groups: causal and non-causal variable nodes. Causal variable nodes significantly impact the central node’s classification, while non-causal variable nodes do not. Causal invariance is then used to enhance the contrastive learning of the drug–target pairs network. Our method demonstrates excellent performance compared with other competitive benchmark methods across multiple datasets. At the same time, the experimental results also show that the causal enhancement strategy can explore the potential causal effects between DTPs, and discover new potential targets. Additionally, case studies demonstrate that this method can identify potential drug targets.

**Availability and implementation:**

The source code of AdaDR is available at: https://github.com/catly/CE-DTI.

## 1 Introduction

The research of drug–target interaction (DTI) prediction is fundamental to the drug discovery and development process, providing critical insights into the molecular interactions between pharmaceutical compounds and their biological targets (Hu *et al.* 2019, Atas Guvenilir and Doğan 2023). Understanding these interactions is crucial for identifying promising drug candidates, elucidating mechanisms of action, and minimizing potential side effects. Furthermore, accurate DTI prediction can reveal potential off-target effects, reducing the risks of adverse reactions (Singh *et al.* 2023). With the growing availability of biological and chemical datasets, advanced DTI models can significantly contribute to precision medicine, paving the way for more effective and personalized treatments for a wide range of diseases (Kim *et al.* 2021).

Traditional biological experiments have played a key role in verifying interactions between drugs and potential targets. These laboratory-based methods provide tangible biological evidence and reveal drug actions at the cellular or molecular level; however, they are often time-consuming and labor intensive (Roalf *et al.* 2016, Jia *et al.* 2024). While in vitro experiments between individual drugs and targets are indispensable, they face numerous challenges when exploring the multiple effects of drugs within biological networks. Researchers often combine different approaches, such as juxtaposing in vitro experiments with computational simulation models, to gain a deeper understanding of drug mechanisms (Naik and Cucullo 2012).

Current computational methods for predicting DTIs can be categorized into four primary types: ligand-based approaches (Wu *et al.* 2024), molecular docking approaches (Shaikh *et al.* 2016), machine learning, and network representation learning methods. Early methods for DTI prediction, such as ligand-based approaches and molecular docking, analyze the structure of drugs that interact with targets using existing pharmacological knowledge, revealing and predicting relationships between chemical structure and pharmacological activity, or simulating the docking of drugs with target structures in three dimensions (Gautam *et al.* 2023). However, the returns from these methods diminish when only a few ligands are known to bind to the target or when the structure of the target is unknown (Srinivasarao and Low 2017).

Traditional machine learning and deep learning algorithms that use the structural and sequence information of targets to predict interactions have gained the attention of researchers because of their ability to quickly screen a vast number of candidates (E *et al.* 2024). These methodologies primarily rely on a hypothesis of homogeneity, which suggests that nodes with similar attributes tend to cluster together, while those with different attributes show divergence. For instance, the DTi2Vec approach projects drugs and targets into a lower-dimensional space, simplifying the interaction prediction process without requiring extensive data mining (Thafar *et al.* 2021). Öztürk *et al.* (2018) introduced the DeepDTA method, which employs convolutional neural networks to independently extract features from drugs and targets, followed by predictions conducted through a deep neural network. Conversely, Lee’s DeepConv-DTI model utilizes a deep belief network for preliminary feature processing (Lee *et al.* 2019). While this method may capture local specific features effectively, it exhibits limitations in cross-domain robustness.

Network representation learning refines traditional machine learning approaches by employing graph neural networks (GNNs) to extract network features for predicting DTIs (Li *et al.* 2022b,c). The DTINet (Luo *et al.* 2017) algorithm, for example, integrates heterogeneous data sources, utilizing low-dimensional features to predict drug−target interaction, while the deepDTnet (Zeng *et al.* 2020) framework explores new drug targets within a multi-source data network. Although the majority of existing approaches extract features by leveraging the structural, sequential, or network information of drugs and targets, these methods often overlook the potential relational modeling between drugs and targets, which restricts their interpretability (Chuang *et al.* 2020). To overcome these limitations and better integrate the potential associations between drugs into the modeling of DTIs, the GCN-DTI model was proposed (Zhao *et al.* 2021), which identifies drug−target interaction by constructing a network based on the multiple attributes of drugs and targets. On the other hand, the IMCHGAN model employs matrix completion and a heterogeneous graph attention network to predict drug−target interaction (Li *et al.* 2021). Although the above methods improve the prediction performance of DTI by fusing multi-source data, they still face challenges such as relying on a lot of prior knowledge, redundant multi-source information, and poor scalability (Jia *et al.* 2021).

The interest ingraph contrastive learning has been rapidly increasing (Li *et al.* 2022a, Fan *et al.* 2022), especially within the field of bioinformatics (Li *et al.* 2023), where its impact is both broad and profound. These methods typically focus on developing graph augmentation strategies, aiming to create rich and diversified representations for each node in the graph (You *et al.* 2020). These strategies work by maximizing the similarity between different representations of the same node and minimizing the similarity between representations of different nodes (i.e. negative samples), with the help of a contrastive loss function. For instance, Yao et al. introduced a framework called SHGCL-DTI, which uses self-supervised contrastive learning to predict the interactions between drugs and targets (Yao *et al.* 2023). The results showed significant improvements in both the predictive power and the generalizability of the model. Building upon this, Yang et al. proposed the GraphCL-DTA model. This model utilizes a graph contrastive learning framework and performs data augmentation in the embedding space, effectively preserving the semantic information of molecular graphs to enhance drug−target binding affinity predictions (Yang *et al.* 2024). These breakthrough methods are highly effective, yet they often overlook the importance of considering causal invariance between nodes. This oversight might lead to misconceptions of non-causal relationships as causal ones during graph representation learning, increasing the model’s confusion and uncertainty.

To address these challenges, we propose a causal enhanced DTI prediction method based on graph generation and multi-source information fusion. Initially, we collected heterogeneous association data between drugs and targets from various sources, alongside detailed drug text descriptions. We use a graph neural network to learn the initial representation of drugs and targets from these data and to achieve multi-source information fusion through an automatic graph generation model. By combining the drug and target representations, we construct a drug−target pair association network, transforming the prediction of drug−target interactions as a node classification problem in graphs. Using causal inference and graph contrastive learning, we eliminate the influence of non-causal confounding factors from surrounding nodes on the central node. Finally, we optimize the performance of the DTI prediction model by jointly refining causal invariance, independence objectives, and classification objectives, all while improving predictive accuracy. Our primary contributions include:

- We introduce a causal-enhanced drug target prediction framework, which enhances the learning representation of drug−target pairs by using the causal invariance of the relationships between drug−target pairs, thus improving the interpretability of the model.
- We conduct numerous comparison experiments on multiple datasets, demonstrating that our proposed CE-DTI model outperforms competitive baseline methods. Additionally, through ablation studies, we verified the model’s rationale, effectiveness, and scalability.
- To our knowledge, this is the first attempt to fuse multi-source information from a graph generation perspective and to leverage causal invariance within graph structures for complex network representation learning, marking a significant advance in the field of high-complexity biological data analysis.

## 2 Materials and methods

**Figure 1.**
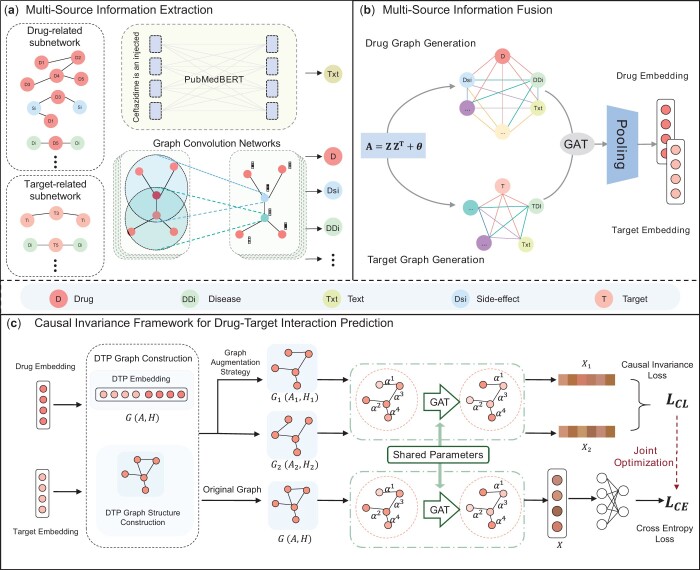
The overall architecture of the CE-DTI model. (a) Extraction of multisource information was accomplished using GAT and pubmedBert, respectively. (b) Multisource information fusion based on graph generation, where the graph structure is constructed using embeddings from multiple information sources, followed by representation learning with a GAT network and weighted fusion. (c) Constructed a DTP network and completed network representation learning, drug–target relationship prediction, and model optimization using causal invariance.

In this section, we elaborate on our proposed **C**ausal **E**nhanced **D**rug-**T**arget **I**nteraction Prediction based on Graph Generation and Multi-Source Information Fusion (CE-DTI) in detail. The overall framework of the model is illustrated in Fig. 1.

### 2.1 Multi-source information extraction

Building upon previous research, our work involves collecting various data from public datasets to construct interaction networks between drugs and targets. These collected datasets mainly comprised four types of entities: drugs (D), targets (T), diseases (Di), and drug side effects (Se). Based on these entities, we established several interaction network, which include: Drug−drug interaction network, drug−disease interaction network, drug−side effect interaction network, drug−chemical structure similarity network, target−disease interaction network, and target−target sequence similarity network, along with a drug−target interaction network serving as a truth benchmark.

To extract features indicative of the interaction between drugs and targets, taking the drug-disease interaction network as an example. We first transform the drug−disease interaction network into a drug−drug homology matrix. This approach not only reveals the associative strengths between drug entities but also considers the transitive associations of drugs through disease entities. After constructing the homology matrix, we apply a graph encoder to perform feature representation learning on this network.

(1) $$\mathrm{Si}m_{\mathrm{DDI}}=M_{\mathrm{DDI}}\times M_{\mathrm{DDI}}^{T},$$

(2) $$\begin{array}{c} \mathrm{Ad}j_{\mathrm{DDI}}(u,v)=\{\begin{array}{l} 1\;\;if\;\mathrm{Si}m_{\mathrm{DDI}}(u,v)>=k_{\mathrm{sim}})\\ 0\;\;if\;\mathrm{Si}m_{\mathrm{DDI}}(u,v)<k_{\mathrm{sim}}) \end{array} \end{array},$$

(3) $$Z_{\mathrm{DDI}}=\mathrm{GraphConv}(\mathrm{Ad}j_{\mathrm{DDI}},E_{D}),$$

where $M_{\mathrm{DDI}}$ represents the adjacency matrix of the drug−disease interaction network, $\mathrm{Si}m_{\mathrm{DDI}}$ represents the degree of association between drugs in terms of disease relationships. When $\mathrm{Si}m_{\mathrm{DDI}}(u,v)$ is greater than a learnable threshold $k_{\mathrm{sim}}$, the drugs *i* and *j* are considered to be associated, $\mathrm{Ad}j_{\mathrm{DDI}}\in R^{n\times n}$ represents the adjacency matrix of drugs under disease interactions, and *n* represents the number of drugs. $E_{D}$ is the initial embedding of drug. $Z_{\mathrm{DDI}}$ represents the features of the drugs in the context of disease associations. With the methods mentioned above, we can acquire other network embeddings of the drugs, such as side effect features $Z_{\mathrm{DSD}}$and drug−drug interaction embeddings $Z_{DD}$.

Additionally, we collected textual description information of drugs from the DrugBank database, which summarizes the key characteristics and properties of the drugs. Integrating the textual information of drugs into our research can enhance our overall understanding of drug properties. Therefore, we chose PubmedBERT (Gu *et al.* 2021), a large-scale biomedical text pre-training model previously developed, as our text information encoder to extract textual information of drugs and obtain the textual embedding vectors of drugs $Z_{\mathrm{TXT}}$.

In summary, we can obtain a multi-source information set for drugs, denoted as $Z_{D}$ = $\left\{Z_{\mathrm{DDI}},Z_{DD},Z_{\mathrm{DSD}},Z_{\mathrm{TXT}}\ldots \right\}$. Similarly, we can also gather a multi-source information set for targets, represented as $Z_{T}$= $\left\{Z_{TT},Z_{\mathrm{TDI}},Z_{\mathrm{Sim}}\ldots \right\}$.

### 2.2 Graph generation and multi-source information fusion

In our method, data sources are treated as network nodes, with edges between nodes representing the relationships between the data. With the use of graph generative models, diverse information can be effectively integrated, such as the chemical properties of drugs, diseases, side effects, etc., reducing reliance on prior knowledge and allowing the network to be updated with new data. Based on these operations, we first obtain the multi-source information embeddings of drugs ($Z_{D}\in R^{n\times p\times d}$) and targets ($Z_{T}\in R^{m\times q\times d}$), where *n* and *m* represent the number of individual drugs and targets, *p* and *q* represent the number of different information sources, and *d* represents the dimension of embedding. For a randomly selected *i*th drug, its multi-source information embedding is $z_{D}^{i}\in R^{p\times d}$. Based on this embedding, we can construct a generative graph’s adjacency matrix, $\mathrm{Generatio}n^{i}$, and the generation process is as follows:

(4) $$Q^{i}=z_{D}^{i}\times z_{D}^{i}{\;}^{T},$$

(5) $$\begin{array}{c} \mathrm{Generatio}n^{i}(u,v)=\{\begin{array}{l} 1\;\;if\;Q^{i}(u,v)>=k\\ 0\;\;if\;Q^{i}(u,v)<k \end{array} \end{array},$$

where $Q^{i}\in R^{p\times p}$ is the product of multi-source information for drug *i*, and the threshold *k* is a learnable value that can be automatically adjusted to an appropriate level through model iterations. Following that, each element of $\mathrm{Generatio}n^{i}$ is determined based on a learnable threshold *k*. For every element in the matrix $Q^{i}$, if its value surpasses *k*, the corresponding position in the matrix $\mathrm{Generatio}n^{i}$ is set to 1, indicating the existence of an association between the respective drug and target pair.

Subsequently, we perform deep representation learning on the generated graphs using GAT. For each drug, we construct an adjacency matrix based on its multi-source information and utilize the attention mechanism of GAT to explicitly distinguish the relative importance of different nodes from various information sources. GAT module ensures that information from different sources can be effectively transmitted and integrated. The embeddings obtained through GAT representation learning are then merged using mean pooling, achieving information fusion without compromising data accuracy. The final embeddings comprehensively represent the feature of each drug:

(6) $$h^{i}=\mathrm{GAT}(\mathrm{Generatio}n^{i},z_{D}^{i})=\sum_{j\in N_{i}}\alpha_{ij}z_{D}^{j},$$

(7) $$H_{D}=\mathrm{MeanPolling}(h)=\frac{1}{p}\sum_{j\in P}h_{j}^{i}$$

where α is the attention weight, which is calculated by formula $\alpha_{ij}=\mathrm{softma}x_{i}(e_{ij})$ and $e_{ij}=\mathrm{LeakyReLU}(a[Wz_{D}^{i}||Wz_{D}^{j}])$. P represents different information sources of drugs. **a** is a learnable transition vector that helps the model dynamically calculate attention weights, enabling the model to focus on important neighboring nodes based on the similarity of node features or other relationships. In light of this, we obtain an embedding matrix based on the multi-source information of drugs and targets, denoted as $H_{D}\in R^{n\times d}$ and $H_{T}\in R^{m\times d}$, respectively.

The drug–target interaction network allows us to match drugs with their targets based on their interactions. We select pairs with explicit interactions, meticulously integrate their information, and concatenate the drug and target embedding vectors end-to-end to create a joint embedding that represents the drug-target interaction. This ensures close linkage and accurately captures the complex drug-target interaction. At the same time, we establish connections between the DTP by analyzing their common features. This not only reflects the natural connection of drugs and targets in the structure but also introduces a biologically meaningful adjacency into the graph $G_{DTP}=(A,H)$, thus constructing a graph that includes an adjacency matrix **A** and a feature matrix $H=[H_{D}||H_{T}]$. The adjacency matrix A of the DTPs network:

(8) $$\begin{array}{c} A=\left[\begin{array}{cccc} f(DTP_{1},DTP_{1}) & \cdots & f(DTP_{1},DTP_{n}) & \\ f(DTP_{2},DTP_{1}) & \cdots & f(DTP_{2},DTP_{n}) & \\ \vdots & \vdots & \cdots & \vdots \\ f(DTP_{n},DTP_{1}) & \cdots & f(DTP_{n},DTP_{n}) & \end{array}\right]=\left(DTP_{ij}\right) \end{array},$$

where the adjacency matrix $A\in R^{N_{\text{DTP}}\times N_{\text{DTP}}}$ represents the relationship of edges between nodes in a graph. The value of $f(DTP_{i},DTP_{j})$ equals to 1, meaning that the two DPPs share some common features, and vice versa.

### 2.3 Causal invariance framework for DTI prediction

In the graph structure $G_{DTP}$, we assume that the neighbors of a node in the graph are divided into two types of variable nodes: The causal variable node set $DTP_{c}$ and the non-causal variable node set $DTP_{s}$. Nodes in $DTP_{c}$ significantly affect the results of the classification task of the central node during graph representation learning, whereas nodes in $DTP_{s}$ do not contribute significantly. Moreover, $DTP_{c}$ and $DTP_{s}$ do not affect each other. To improve the representativeness of the graph structure and enable the model to extract causal variables more effectively, we introduce a random augmentation strategy for the graph structure. Before encoding the graph structure, we mask certain dimensions of node embeddings or the associations between nodes in the original graph structure, thereby introducing perturbations to the graph structure before each training round. Under this strategy, we construct two augmented graphs:

(9) $$G_{\mathrm{aug}1}=(A_{\mathrm{aug}1},H_{\mathrm{aug}1});G_{\mathrm{aug}2}=(A_{\mathrm{aug}2},H_{\mathrm{aug}2})$$

During this augmentation process, we have expanded the nodes and features for two graphs: In $G_{\mathrm{aug}1}$, the causal variable node set is labeled $DTP_{c1}$ and the non-causal variable node set as $DTP_{s1}$; and in $G_{\mathrm{aug}2}$, the causal variable node set as $DTP_{c2}$ and the non-causal variable node set as $DTP_{s2}$. Ideally, no matter how the non-causal variable set changes, the model should maintain the consistency of the causal variable nodes around the central node in both graphs, ensuring that $DTP_{c1}$ = $DTP_{c2}$ =$DTP_{c}$. After going through the representation learning process, the model should be better at identifying and extracting features that remain stable and unchanged under different augmentation strategies.

However, the stochastic augmentation strategy cannot ensure the stability of the causal set after graph perturbation. This strategy may integrate non-causal information into DTP node embeddings, and when non-causal variables change, we want the model to accurately extract causal information and filter out non-causal interference. Therefore, the key to our augmentation strategy is to selectively perturb the non-causal variable set while maintaining the stability of the causal variable set:

(10) $$P^{do(DTP_{s}=DTP_{si})}(Y|DTP_{c})=P^{do(DTP_{s}=DTP_{sj})}(Y|DTP_{c}),$$

where the notation $do(DTP_{s}=DTP_{s*})$ signifies an intervention on the non-causal factors $DTP_{s}$, the model is encouraged to focus solely on information extracted from the causal factors $DTP_{c}$, while disregarding any inconsequential information.

Recently, in the study by Liu *et al.*, it is mentioned that by perturbing the structure of the initial graph, the frequency intensity of node information in its spectrum can be changed. Different frequency components carry different levels of information that have varying impacts on the graph structure, where high-frequency components often exhibit greater informational disparities than low-frequency components (Liu *et al.* 2022). Consequently, we have reason to consider *low-frequency components* as invariant causal information, which remains consistent across different views of the graph. Considering that we cannot rely on label information during the graph augmentation process, we need to adopt a new method to maintain the invariance of causal information, while allowing for the perturbation of non-causal information. Therefore, we modify the objective function of the model, targeting causal relationship variables (i.e. causal effects), which should satisfy the condition of consistent causal relationships under different non-causal perturbations. In other words, we aim to achieve:

(11) $$CE(DTP_{c},DTP_{s}=DTP_{si})=CE(DTP_{c},DTP_{s}=DTP_{sj})$$

where CE(*) refers to the causal effect, and $DTP_{si}$ and $DTP_{sj}$ represent the sets of non-causal information under two different perturbations, respectively.

To capture the consistency of representation learning in augmented graphs, we employ graph attention networks to aggregate neighbor information, integrating node features and structural characteristics. Furthermore, we encourage the model to focus on learning invariant causal relationships through the loss function, while ignoring or minimizing the impact of non-causal information introduced by graph augmentation:

(12) $$X_{1}=\mathrm{GAT}(A_{\mathrm{aug}1},H_{\mathrm{aug}1}),$$

where $A_{\mathrm{aug}1}$ is the adjacency matrix of the graph structure, $H_{\mathrm{aug}1}$ is the initial embedding of the graph structure.

### 2.4 Model optimization

Based on the above calculation, we can obtain embeddings of two augmented graphs, $X_{1}$ and $X_{2}$. In order to encourage the embeddings to remain invariant across augmented graphs, we propose a learning objective which enforces $X_{1}$ and $X_{2}$ to follow the same mean and variance along their dimensions, thereby imposing a regularization on the embedding representations in a statistical sense. The learning objective can be formalized as

(13) $$\underset{g}{\min}\sum_{i}{\left\|X_{1}^{i}-X_{2}^{i}\right\|}_{2}^{2},\;s.t.\mathrm{Std}(X_{1}^{i})=s.t.\mathrm{Std}(X_{2}^{i})=\lambda ,$$

where $X_{1}^{i}$ and $X_{2}^{i}$, respectively, represent the *i*th dimension of the two embedding matrices, and s.t.Std(*) represents the standard deviation. The first term encourages the means of the two embedding matrices to be equal along the same dimension, and the second term promotes the standard deviation to be close to λ.

We further normalized the dimensions of the embedding matrix and denoted it with $\tilde{X}$ (note that ${\left\|{\tilde{X}}^{i}\right\|}^{2}$ = 1), and therefore $min_{g}\sum_{i}{\left\|{\tilde{X}}_{1}^{i}-{\tilde{X}}_{2}^{i}\right\|}^{2}$ can be replaced by calculating the squared Euclidean distance between embeddings ${\tilde{X}}_{1}^{i}$ and ${\tilde{X}}_{2}^{i}$ can be turned into maximizing their inner product in each dimension, which is $max_{g}\sum_{i}{\tilde{X}}_{1}^{i}\cdot {\tilde{X}}_{2}^{i}$, and $s^{i}$ represents the standard deviation in each dimension before the normalization of the original standard deviation. Minimizing $\sqrt{{\left\|s_{i}-\lambda \right\|}_{2}^{2}}$ makes the standard deviation close to λ. The causal optimization we propose is as follows:

(14) $$L_{CL}=-\alpha {\tilde{X}}_{1}^{i}\cdot {\tilde{X}}_{2}^{i}+\beta \sum_{i}\left(\sqrt{{\left\|s_{1}^{i}-\lambda \right\|}_{2}^{2}}+\sqrt{{\left\|s_{2}^{i}-\lambda \right\|}_{2}^{2}}\right),$$

where α and β are hyperparameters that control the importance of each term in the loss. λ represents the desired standard deviation per dimension.

Finally, a GAT with shared parameters with the augmented graph is utilized to learn feature representations of the original graph, which are then classified by a downstream classifier based on the embeddings:

(15) X=GAT(A,H),

where ***A*** and H are the adjacency matrix and the embedding matrix of $G_{DTP}$.

Similar to previous studies, we modeled the drug-target interaction prediction task as a binary classification task, aiming to identify whether there is an interaction between the drug and the target, that is, *Y* belongs to {0,1}. The loss function defined between the predicted results and the true value label $L_{CE}$ is as follows:

(16) $$L_{CE}=-\sum_{i=1}^{N_{\mathrm{DTP}}}\left[y_{i}\;\text{log}\;\pi \left(x_{i}\right)+\left(1-y_{i}\right)\;\text{log}\;\left(1-\pi \left(x_{i}\right)\right)\right],$$

where π(x)=P(Y=1|X) and y∈{0,1}.

Finally, the optimization objective of the model consists of two parts: The classification cross-entropy loss and the contrastive loss.

(17) $$L=L_{CE}+L_{CL}.$$

## 3 Results

In this section, we conduct a series of experiments to investigate the applicability and efficacy of our model. Specifically, we aim to address the following research questions: (i) Does the CE-DTI framework outperform state-of-the-art methods in DTI prediction tasks? (ii) What role does the introduced causal-enhanced mechanism play in DTI prediction tasks? Can it provide additional insights or explanations for the model’s predictions? (iii) Is the implementation of graph generation and multi-source information fusion within the CE-DTI model necessary? (iv) Does the model offer potential insights into mining drug-target interaction?

### 3.1 Datasets

To assess the performance of our model, we have conducted evaluations on two widely recognized and utilized datasets: the Luo dataset (Luo *et al.* 2017) and the Zheng’s dataset (Zheng *et al.* 2018).

- *Luo’s dataset* (Luo *et al.* 2017): The Luo’s dataset includes four types of nodes (drugs, targets, diseases, and side effects) along with eight types of associations between these nodes. Using these associations, we constructed four heterogeneous interaction networks (Drug–Target, Drug–Disease, Drug–Side Effect, and Target–Disease networks) and four homogenous similarity networks (Drug Chemical Structure Similarity network, Target Sequence Similarity network, Drug Interaction network, and Target Interaction network), involving 708 drugs, 1512 targets, 4192 side effects, and 5603 disease nodes.
- *Zheng’s dataset* (Zheng *et al.* 2018): The Zheng’s dataset encompasses 1094 drugs and 1556 targets, along with 11 819 experimentally verified interactions. This dataset also includes 881 chemical structural pieces of information and 738 drug substituents information from DrugBank, as well as chemical substructure information from PubChem. In addition, it integrates 4063 drug side effect records from SIDER and target Gene Ontology (GO) terms from EMBL-EBI.

### 3.2 Baseline

To benchmark the performance of our model, we employed the following well-established competitive methods for DTI prediction:

- **DTINet** (Luo *et al.* 2017): This model generates low-dimensional feature vectors for drugs and targets through a network integration approach, focusing on finding the optimal mapping from drug space to target space. DTINet sets a strong baseline for DTI prediction by integrating multiple biological networks and mapping the potential associations between drugs and targets.
- **IMCHGAN** (Li *et al.* 2021): An end-to-end DTI prediction system that not only extracts features of drugs and targets from a heterogeneous network but also leverages inductive matrix completion techniques for prediction. IMCHGAN is adept at capturing complex drug−target relationships and effectively utilizing existing interaction data.
- **GCN-DTI** (Zhao *et al.* 2021): GCN-DTI incorporates the associations within drug–protein pairs by constructing a DPP network based on multiple drugs and proteins. Each DPP serves as a node, and the associations between DPPs are represented as edges. The model then utilizes a GCN to learn the features of each DPP, and a deep neural network is employed to predict the labels.
- **EEG-DTI** (Peng *et al.* 2021): Based on heterogeneous GCN, this model constructs an end-to-end learning framework. EEG-DTI employs deep learning techniques to capture relational data within biological heterogeneous networks and predicts DTI by computing the inner product of learned feature representations.
- **SHGCL-DTI** (Yao *et al.* 2023): This model combines the semi-supervised learning task of DTI prediction with graph contrastive learning. It optimizes the representation learning process by generating representations of nodes under different views and defining positive and negative sample pairs. This strategy, merging similarity learning with contrastive learning, aims to better capture the interactions between drugs and targets.

### 3.3 Main results

**Figure 2.**
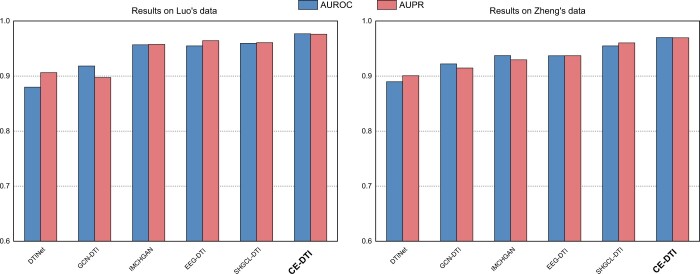
Comparison of the AUROC and AUPR values between the CE-DTI model and baseline methods, demonstrating that our model outperforms the baselines on Luo’s and Zheng’s datasets.

We conducted a comprehensive comparison for DTI prediction tasks by evaluating our model on two datasets, comparing it with five mainstream methods. The corresponding area under the receiver operating characteristic curve (AUROC) and the area under the precision-recall curve (AUPR) results can be seen in Fig. 2.

Comparative analysis shows that the methods using graph neural network, including IMCHGAN (Li *et al.* 2021), GCN-DTI (Zhao *et al.* 2021), EEG-DTI (Peng *et al.* 2021), and SHGCL-DTI (Yao *et al.* 2023), and our model, exhibit superior performance compared to DTINet (Luo *et al.* 2017). This underscores the efficacy of deep graph neural networks in learning features of drugs and targets; they use node attributes and node interactions to update node information, thereby obtaining more accurate feature representations and enhancing the model’s generalization capabilities. At the same time, the performance of methods that integrate multi-source information in a targeted manner is slightly superior to GCN-DTI and EEG-DTI, which may be attributed to the heterogeneous nature of the information. Different sources of information are distributed in different spaces and handling them with the same encoder could lead to information loss, which in turn affects the generalization ability of the model. SHGCL-DTI and our model, which involve perturbations to the graph structure during processing, show superior performance that could be associated with the enhanced noise-resistant capability of the model after undergoing perturbation training.

### 3.4 Ablation study

In our ablation study, we aimed to examine the necessity of each module within the CE-DTI model. By systematically removing key components from the model, we evaluated the contribution of the remaining parts to the overall performance of the model. Specifically, we incrementally eliminated modules from the CE-DTI model and trained and predicted with the remaining model composition. In the experiments, we defined several strategies: Firstly, the “w/o textual information source” strategy validated the impact of textual information on model performance by excluding text description embeddings from the model’s multi-source information input. Secondly, the “w/o causal representation” strategy removed the causal invariance loss component from the model, retaining only the predictive cross-entropy loss. Then, the “w/o graph-based multi-source information” strategy abandoned the graph generation approach and instead used a method based on meta-paths to integrate multi-source data. Lastly, the “feature-based graph construction” strategy utilized embedding inner products instead of topological structures when constructing the DTP network.

Based on the experimental data in Table 1, we can make the following conclusions: When the textual information source was removed, the model’s generalization performance declined, indicating that textual information is likely rich in descriptive data about drug molecular properties. This information can help the model more accurately predict interactions between drugs and targets, as it provides more nuanced drug characteristics insights than structural information alone. After the removal of the causal representation, the model’s performance decreased by 2%, suggesting that the causal framework plays a role in reducing noise and irrelevant data interference, and in reinforcing the extraction of causal information. When the integration of multi-source information shifted from a graph generation method to a method based on meta-paths, the model’s performance significantly dropped by about 6%, possibly because the graph generation method can more finely mimic bio-network changes and adaptability, providing better capture of new information and uncertainties. Although meta-path methods can capture indirect relationships and patterns between entities, they might not be sufficient to grasp more complex or evolving network structure features compared to graph generation methods. If the feature-based graph construction method replaced the topology-based method, the model’s generalization ability was also affected. This could be because using a feature-based graph construction method might cause the model to be biased towards processing and recognizing local feature interactions, and not adequately capturing and understanding the complex network relationships between drugs and targets.

**Table 1.** Results of ablation study on Luo’s dataset.

Method	AUROC	AUPR
w/o textual information source	0.9698	0.9701
w/o causal representation	0.9677	0.9674
w/o graph-generated multi-source information	0.9289	0.9302
feature-based graph construction	0.9549	0.9651

CE-DTI	0.9768	0.9760

### 3.5 Causal invariance analysis

Experiments have shown that optimization for causal invariance has a positive effect on model training. Under this optimization framework, the application of graph augmentation strategies plays an extremely important role in simulating the uncertainty of biological processes, improving the model’s generalization ability, and adapting to data disturbances. To further verify whether the causal invariance framework can provide effective information for node classification, we selected nodes with minor changes in two augmented graphs as the training set to test the model’s generalization performance. The experiment proved that even when only a small number of causal nodes are used as the training set, good model generalization can be achieved.

**Figure 3.**
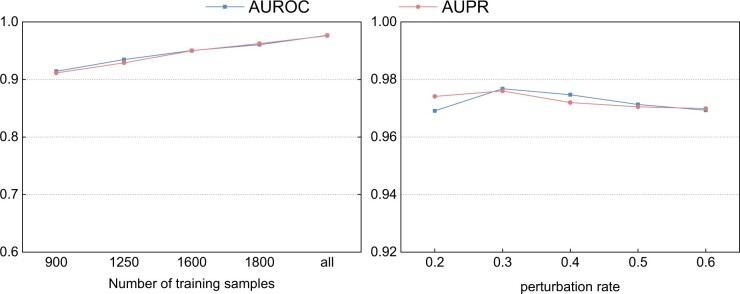
Causal invariance analysis experiment. The left side shows the results of training the model with different numbers of causal nodes, and the right side presents the analysis results of the causal perturbation parameters.

On the other hand, we also attempted to explore the impact of disturbance factors on the experimental results and the robustness of the methods used. In the experiment, we set five different levels of disturbance rates [0.2, 0.3, 0.4, 0.5, 0.6] to augment the graph. The experimental results, as shown in Fig. 3, where each point represents the result of graph structure disturbance caused by different disturbance rates. The choice of disturbance rate directly affects the model’s learning process. A lower disturbance rate (such as 0.2) may not provide enough variation, while a higher disturbance rate (such as 0.5 and above) may destroy the core structure of the graph, causing the model to fail to identify the real drug-target relationship.

### 3.6 Analysis of the effectiveness of graph generation

**Figure 4.**
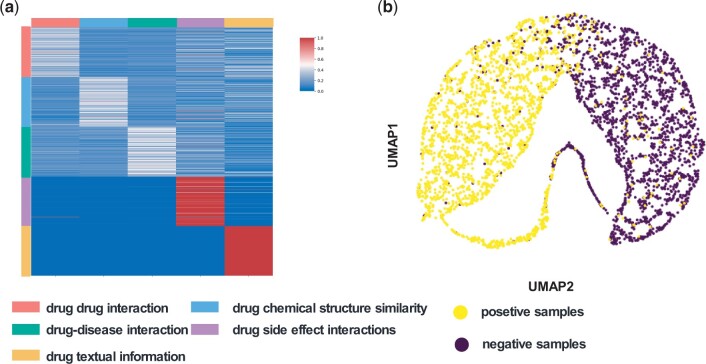
Information integration analysis based on graph generation. (a) Visualization of the weights of the multi-source information association network generated by the graph. (b) Visualization of DTP embeddings for positive and negative examples.

In this section, we analyze the graphs generated by the multi-source information fusion method based on graph generation to assess the effectiveness of the proposed graph generation approach. Specifically, we conducted experiments on Luo’s dataset, where we transformed the multi-source information embeddings of each drug into weights for subgraphs and visualized them. As shown in Fig. 4, this illustrates the connection weights between each information source and the remaining sources. From the results in Fig. 4a, it can be seen that the text information source and drug side effect information source carry greater weight in the generated graph, highlighting the importance of these two features in the model’s performance and emphasizing the necessity of integrating these sources for optimal results. Ablation experiment results support this conclusion, showing a decrease in the model’s generalization performance after the text information source is removed, which further suggests the rich value of the descriptive data about drug molecular properties contained in the text source.

To evaluate which type of multi-source information has the most significant impact on the model’s generalization performance, we systematically removed each source of information and trained and tested the model using the remaining data. As shown in Table 2, the removal of drug side effect information resulted in a substantial decline in the model’s generalization performance. This result is consistent with Fig. 4, indicating that drug side effect information carries substantial weight in the model and plays a critical role in its overall performance.

**Table 2.** Results of multi-source information ablation study.

Method	AUROC	AUPR
w/o drug−drug interaction network	0.9679	0.9701
w/o drug−disease interaction network	0.9680	0.9723
w/o drug−side effect interaction network	0.9582	0.9594
w/o drug−chemical structure similarity network	0.9624	0.9628
w/o target−target interaction network	0.9398	0.9461
w/o target−disease interaction network	0.9700	0.9723
w/o target−target sequence similarity network	0.9464	0.9487
CE-DTI	0.9768	0.9760

Furthermore, we utilized the Umap method to visualize the spatial distribution of positive and negative node embeddings in the DTP network after multi-source information integration. Figure 4b clearly shows the distinct distributions of the two classes of nodes, confirming the effectiveness of our proposed information integration method.

### 3.7 Case study of CE-DTI in discovering potential targets

In this study, we utilized the proposed model CE-DTI to predict potential targets for drug repositioning and validated these predictions through molecular docking. We focused on the potential targets of Pergolide to explore its possibilities for drug repositioning. Pergolide is a drug that mimics the action of dopamine in the brain and is primarily used to treat diseases related to dopamine deficiency (Kimberg and D’Esposito 2003), such as Parkinson’s disease. Its mechanism of action is mainly related to the nervous system, and it acts as an agonist to specific dopamine receptors (Kaur *et al.* 2020).

**Figure 5.**
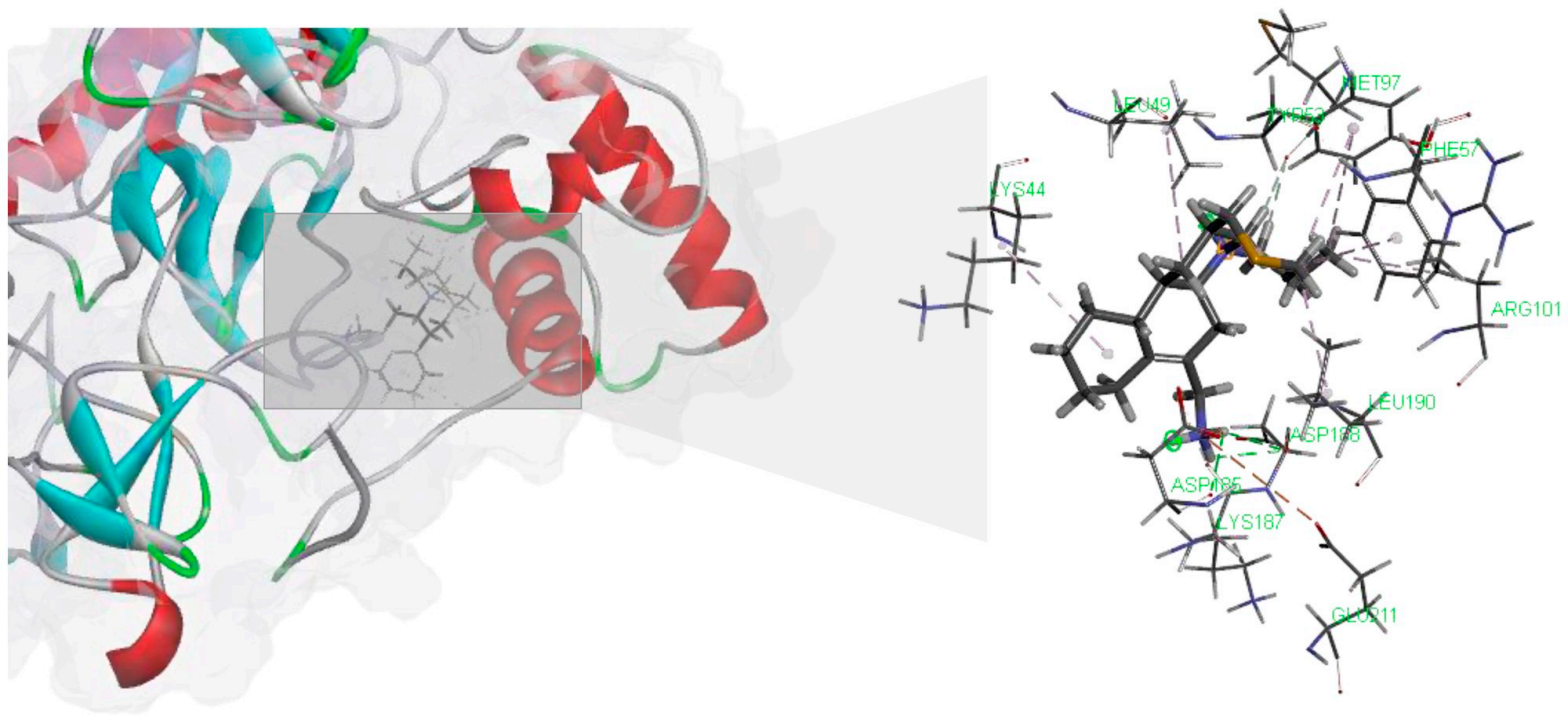
Potential docking sites between Pergolide and MMP2 identified by the LibDock method.

According to the predictions made by the our model, we were able to confirm the accuracy of seven positive target samples for Pergolide in the test set. Notably, we discovered that the drug had an exceptionally high interaction score with MMP2 (P08253). To further validate this finding, we employed the LibDock (Rao *et al.* 2007) molecular docking program and identified four potential docking sites on this target with a high docking score of 100.026, as shown in Fig. 5. This suggests the possibility of an interaction between Pergolide and MMP2. These discoveries, along with other prediction results, will be detailed in our forthcoming dataset publication.

### 3.8 Cold-start and model robustness analysis

Previous experiments have demonstrated that DTI prediction based on multi-source information fusion can significantly enhance prediction performance. However, the generalization ability of most models may significantly decline when the known interaction between drugs and targets are limited, or when a new drug or target is introduced. Therefore, to ensure the robustness of the experimental results, we first tested the model under conditions of data imbalance. We designed a positive and negative sample selection strategy by treating all data as positive samples and randomly selecting a number of negative samples from the negative data set. We tested the model’s generalization performance under positive-to-negative sample ratios of 1:1, 1:2, and 1:3, as shown in Table 3. The results indicate that the model maintains good generalization performance even in the presence of sample imbalance.

**Table 3.** Results of imbalanced positive-to-negative ratio experiments.

Ratio	AUROC	AUPR
1:1	0.9768	0.9760
1:2	0.9612	0.9443
1:3	0.9572	0.9391

Subsequently, to evaluate the model’s performance in real-world scenarios, we conducted cold-start experiments for both drugs and targets. By partitioning the dataset so that certain drugs or targets were completely excluded from training, we evaluated the model’s predictive ability on these unseen drugs or targets. The CE-DTI model was compared with two different heterogeneous information aggregation methods as baseline models. The results showed that while the EEG-DTI model achieved a slightly higher AUROC score in certain instances (see Tables 4 and 5), this may be due to its focus on threshold optimization within its heterogeneous GCN framework. However, the CE-DTI model exhibited superior accuracy overall, which is more beneficial for DTI prediction, as it demonstrates a stronger ability to correctly predict a wide range of interactions, making it more reliable for real-world applications. These experimental results suggest that the CE-DTI model maintains good predictive ability in handling data imbalance and cold-start issues, demonstrating high reliability and practical utility.

**Table 4.** Drug cold-start experiments on the Luo’s dataset.

Method	ACC	AUROC
IMCHGAN	0.8466	0.9503
EEG-DTI	0.8847	0.9594
CE-DTI	0.8988	0.9548

**Table 5.** Target cold-start experiments on the Luo’s dataset.

Method	ACC	AUROC
IMCHGAN	0.8786	0.9394
EEG-DTI	0.8957	0.9449
CE-DTI	0.9189	0.9404

## 4 Conclusion

In this article, we propose a causal enhanced method for drug-target interaction (CE-DTI) prediction. Our model, when integrating multi-source information of drugs and targets, not only preserves the diversity of the data but also maintains the structural features of the graph. Our methods significantly enhances the quality of the drug and target embeddings. Furthermore, we have taken into account the potential causal relationships among the nodes in the graph and have introduced the principle of causal invariance as a constraint. It helps to reduce the interference of non-causal information during model perturbations while preserving important causal information. These factors together have contributed to the outstanding performance of our model across various datasets.

To validate the stability of the proposed model, we compared CE-DTI with five of the most advanced methods currently available. The comparison showed at least 2% improvement in the AUROC and AUPR evaluation metrics. Ablation studies further demonstrated the core role of our model in the task of predicting drug-target interactions. Additionally, through case studies, we demonstrated the effectiveness of the model in identifying potential drug targets. It not only confirms the predictive power of the model but also provides a new direction for the research of targeted drug therapy.

Although CE-DTI has shown promising results, there are still several limitations that need to be addressed. First, the model is highly dependent on high-quality multi-source information embeddings. When applied to incomplete or noisy datasets, its performance may significantly degrade. Therefore, future research could focus on developing more robust methods to handle missing or unreliable data, such as utilizing advanced data imputation techniques or incorporating uncertainty quantification to enhance the model’s resilience to noise. Furthermore, while CE-DTI has demonstrated outstanding performance in identifying potential drug targets, its application in other related tasks has not yet been fully explored. For instance, predicting drug-drug interactions or multi-target drugs remains a critical challenge in drug discovery. Extending the model to these areas could potentially provide a more comprehensive and effective tool for drug discovery and development.

## Data Availability

Experimental data sets and experimental codes can be found in https://github.com /catly/CE-DTI.
